# Design of a randomised controlled hybrid trial of nintedanib in patients with progressive myositis-associated interstitial lung disease

**DOI:** 10.1186/s12890-024-03314-0

**Published:** 2024-10-30

**Authors:** Rohit Aggarwal, Chester V. Oddis, Daniel I. Sullivan, Siamak Moghadam-Kia, Didem Saygin, Daniel J. Kass, Diane C. Koontz, Peide Li, Craig S. Conoscenti, Amy L. Olson, on behalf of the MINT investigators

**Affiliations:** 1 University of Pittsburgh School of Medicine, Pittsburgh, PA USA; 2 Boehringer Ingelheim Pharmaceuticals, Inc, Ridgefield, CT USA; 3 Present Address: Avalyn Pharmaceuticals, Inc, Seattle, WA USA

**Keywords:** Clinical trial, Connective tissue diseases, Dermatomyositis, Polymyositis, Interstitial fibrosis, Pulmonary fibrosis

## Abstract

**Background:**

The Myositis Interstitial Lung Disease Nintedanib Trial (MINT) is a hybrid trial, which is enrolling patients both at local sites and remotely via a decentralised site. The trial will investigate the efficacy and safety of nintedanib in patients with progressive myositis-associated interstitial lung disease (MA-ILD).

**Methods/Design:**

MINT is an exploratory, prospective randomised placebo-controlled trial. Eligible patients will have myositis and evidence of fibrosing ILD on high-resolution computed tomography (HRCT), be taking standard of care medications for myositis, and meet criteria for ILD progression within the prior 24 months based on decline in FVC, worsened fibrosis on HRCT, and/or worsened dyspnoea. Patients will be randomised 1:1 to receive nintedanib 150 mg twice daily or placebo for 12 weeks then open-label nintedanib for 12 weeks. Patients will be enrolled at local sites and a decentralised site. Most study visits will be completed remotely using telemedicine or digital health technologies. The primary endpoint is the change in Living with Pulmonary Fibrosis (L-PF) questionnaire dyspnoea domain score at week 12. Other endpoints include changes in other L-PF questionnaire domains, lung function, imaging, and physical activity, and assessment of adverse events. Data collected using remote versus clinic enrolment, and using home versus clinic spirometry, will be compared.

**Discussion:**

MINT is an innovative, hybrid trial that will evaluate the effects of nintedanib on symptoms, quality of life, and ILD progression in patients with progressive MA-ILD and provide valuable information on the utility of decentralised recruitment and remote data collection in clinical trials.

**Trial registration:**

Clinicaltrials.gov NCT05799755 (date of registration: 05/04/2023).

**Supplementary Information:**

The online version contains supplementary material available at 10.1186/s12890-024-03314-0.

## Plain language summary

- The term “myositis” describes a group of diseases in which an overactive immune system causes damage to muscles.
- Some patients with myositis develop lung fibrosis (scarring of the lungs). Lung fibrosis may get worse over time, leading to loss of lung function and to symptoms such as shortness of breath and cough.
- Nintedanib is a drug used to treat lung fibrosis.
- A trial known as “MINT”, which stands for the “Myositis Interstitial Lung Disease Nintedanib Trial”, is looking at the effects of nintedanib compared to placebo in patients with myositis who have lung fibrosis that has got worse over the previous two years.
- The trial will look at the effects of nintedanib on symptoms, lung function and other measures of the severity of lung fibrosis.
- The design of “MINT” is unusual in that patients can be enrolled into the trial virtually, from anywhere in the US, as well as at sites such as hospitals, and most of the data will be collected remotely using devices that patients can use at home.
- The results of this trial will improve our understanding of the effects of nintedanib in patients with myositis and lung fibrosis and provide important information on the remote collection of data in clinical trials.

## Background

Myositis comprises a group of autoimmune disorders characterised by weakness and inflammation of the muscles [1]. A proportion of patients with myositis develops interstitial lung disease (ILD) [2–5], which is associated with cough, dyspnoea, fatigue, reduced quality of life, and premature mortality [6–8]. Patients with anti-synthetase antibodies are at greater risk of developing ILD than patients with other myositis autoantibodies [8]. In clinical practice, myositis-associated ILD (MA-ILD) is usually treated with glucocorticoids and/or other immunosuppressive therapies [9–11]. A guideline issued by the American College of Rheumatology and American College of Chest Physicians conditionally recommended mycophenolate, azathioprine, rituximab, cyclophosphamide, Janus kinase inhibitors, calcineurin inhibitors, or short-term glucocorticoids as first-line treatment options for idiopathic inflammatory myopathies including MA-ILD [12].

Despite treatment, some patients with MA-ILD develop progressive pulmonary fibrosis characterised by increasing radiological fibrosis, decline in lung function, and premature mortality [3, 4, 13–16]. Nintedanib is a tyrosine kinase inhibitor that inhibits processes fundamental to the progression of pulmonary fibrosis [17, 18]. In the recent guideline issued by the American College of Rheumatology and American College of Chest Physicians, nintedanib received a conditional recommendation for use in patients whose MA-ILD had progressed despite first-line treatment [12]. A guideline issued by international respiratory societies for the treatment of progressive pulmonary fibrosis in patients with diagnoses other than idiopathic pulmonary fibrosis (IPF) also provided a conditional recommendation for the use of nintedanib in patients who have “failed standard management” [19]. In the randomised, placebo-controlled INBUILD trial in patients with progressive pulmonary fibrosis, nintedanib reduced the decline in forced vital capacity (FVC) over 52 weeks by 57%, with an adverse event profile characterised mainly by gastrointestinal events [20–22], similar findings to those previously observed in patients with IPF [23] and systemic sclerosis-associated ILD [24]. The INBUILD trial was not powered to study patients with specific diagnoses, but no heterogeneity was detected in the effect of nintedanib across subgroups by diagnosis [25, 26], or based on the introduction of immunomodulatory therapies during the trial [27]. Data collected using the Living with Pulmonary Fibrosis (L-PF) questionnaire suggested that nintedanib lessened the symptoms and impact of ILD [28]. However, nintedanib is not commonly used in patients with MA-ILD, likely due to a lack of clinical data supporting its safety and efficacy in this specific population; only two patients in the INBUILD trial had a diagnosis of polymyositis‐associated ILD or antisynthetase syndrome noted on the case report form [26].

The Myositis Interstitial Lung Disease Nintedanib Trial (MINT) was designed to evaluate the effects of nintedanib in patients with progressive MA-ILD taking standard of care therapy. MINT has a novel hybrid design that includes both traditional clinical sites and a decentralised site, and most study visits will be conducted remotely. This will enable evaluation of the utility of decentralised recruitment and remote data collection, and of novel measures such as physical activity monitors and home spirometry. In this manuscript, we describe the key design elements of this innovative trial.

## Methods/design

### Study design and participants

**Fig. 1:**
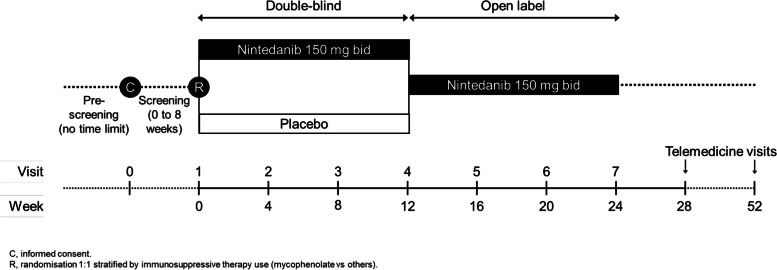
Design of MINT

MINT is an exploratory, multi-centre, prospective, randomised, double blind, placebo-controlled trial. Eligible patients will be randomised 1:1 to receive nintedanib 150 mg twice daily (bid) or placebo stratified by local vs decentralised site and by use of immunosuppressive therapy (mycophenolate mofetil vs others). Patients will receive blinded treatment for 12 weeks, after which all patients will receive open-label nintedanib for 12 weeks (Fig. 1). Treatment interruptions for ≤ 4 weeks and dose reductions to 100 mg bid will be allowed to manage adverse events. Specific recommendations will be provided to the investigators for the management of diarrhoea and hepatic enzyme elevations (Additional file 1). Patients who prematurely discontinue trial medication will be asked to complete all scheduled visits.

### Recruitment, consent, and screening

**Fig. 2:**
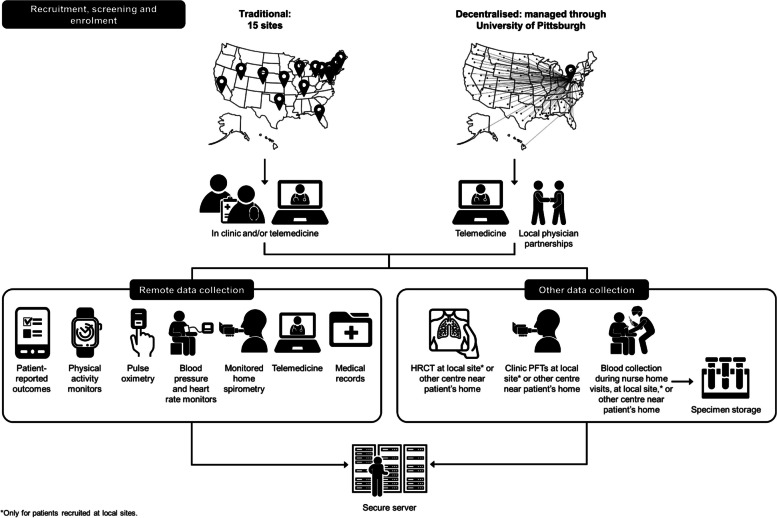
Flow through decentralised recruitment and data collection in MINT

**Fig. 3:**
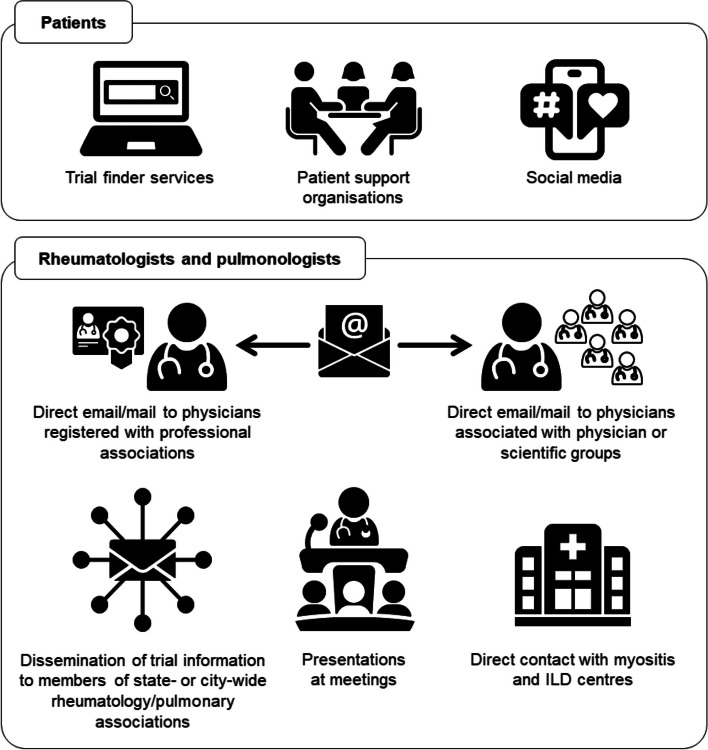
Methods used to approach patients and physicians for decentralised recruitment to MINT

The trial will use two methods of recruitment: at local sites and decentralised (Fig. 2). Patients will be recruited conventionally at 15 sites through clinic referrals, local registries, advertisements on flyers and websites, and mailings/emails to local physicians. For these patients, pre-screening and consent can be conducted at the local site, via telemedicine, or a combination of both. In addition, patients from anywhere in the USA will be recruited using a decentralised approach, managed through the University of Pittsburgh. Patients and physicians will be approached using a number of methods including via social media and patient/physician organisations (Fig. 3). Patients will be directed to a website providing detailed information about the trial, a consent form, and a link to a preliminary pre-screening questionnaire. After completing this questionnaire, they will be directed for pre-screening consent and release of medical information. Approval will be obtained from the patient’s treating physician. Medical records will be reviewed by a trial investigator. Consent for screening will be obtained via telemedicine and a screening visit will then be arranged.

For all patients, screening will be conducted via telemedicine and using digital health technologies at the patient’s home, except for high-resolution computed tomography (HRCT) scans (if one has not been done in the prior 3 months) and clinic pulmonary function tests (PFTs), which will be performed at a centre near the patient’s home. For the blood sampling needed for laboratory assessments, a research nurse will visit the patient’s home. Patients recruited via a local site will also have the option to have some screening assessments at the local site.

The trial protocol was approved by an ethics review board at the University of Pittsburgh School of Medicine (STUDY22090057). The trial will be conducted in accordance with the International Conference on Harmonisation Guideline for Good Clinical Practice, Declaration of Helsinki principles, and local regulatory, ethical, and legal requirements. All patients will provide written informed consent. Results will be monitored by an independent Data Safety Monitoring Board. An independent medical monitor will ensure required activities are performed according to standard operating procedures and direct the co-ordinating team on conduct and reporting. The study is registered on clinicaltrials.gov (NCT05799755; date of registration: 05/04/2023). Results will be published in a peer-reviewed publication.

### Randomisation and drug dispensing

Randomisation and dispensing of trial drugs will be coordinated through a central pharmacy. Once a patient passes screening, the central pharmacy will complete the randomisation form and trial drugs will be shipped directly to the patient’s home. In the case of early discontinuation, any remaining drug will be shipped back to the central pharmacy.

### Visits and measurements

Most study visits will be completed remotely (Fig. 2). Physician and trial co-ordinator visits will be conducted via telemedicine. Patients will be provided with various monitoring devices: blood pressure and heart rate monitor, home spirometer, pulse oximeter, and physical activity monitor wristwatch. Patients will receive training on how to use these devices, including home spirometry training from a PFT technician via telemedicine during screening. Patients will complete patient-reported outcome questionnaires on tablets.

Two types of home spirometry will be conducted. Twice a week, patients will perform home spirometry guided by an avatar on a tablet, but not supervised by a PFT technician. In addition, at baseline and every 4 weeks thereafter, patients will perform home spirometry guided by a PFT technician via telemedicine. Spirometry measurements will be performed according to American Thoracic Society/European Respiratory Society guidelines [29]; patients will be asked to repeat the spirometry if these requirements are not met.

For patients recruited via the decentralised approach, clinic PFTs and HRCT will be performed at a centre near the patient’s home. Patients recruited via a local site will have clinic PFTs and HRCT at the local site or at another centre near their home. After screening, clinic PFTs will be performed at weeks 12 and 24, and an HRCT will be completed at week 24.

For patients recruited via the decentralised approach, blood draws will be made by a research nurse during home visits or at a centre near the patient’s home. Patients recruited via a local site will have blood draws at the local site or at another centre near their home.

### Patients

Eligible patients will be aged ≥ 18 years, with a diagnosis of myositis based on the 2017 European League Against Rheumatism/American College of Rheumatology classification criteria [1] or the presence of myositis autoantibodies (Additional file 2), fibrosing ILD (reticular changes, traction bronchiectasis and/or honeycombing) on an HRCT scan taken within the prior 12 months, and an FVC > 40– ≤ 80% predicted. Patients must meet ≥ 1 of the following criteria for ILD progression at any time within the prior 24 months: relative decline in FVC ≥ 10% predicted; relative decline in FVC ≥ 5– < 10% predicted with worsened dyspnoea; relative decline in FVC ≥ 5– < 10% predicted with worsened fibrosis on HRCT; worsened dyspnoea and worsened fibrosis on HRCT. Patients must be taking 1 or 2 standard of care therapies (glucocorticoid and/or other immunosuppressant listed in Additional file 2) at a stable dose. For patients taking 2 therapies, ≥ 1 must be a non-glucocorticoid. Patients who have taken immunosuppressants other than those listed in Additional file 2 will be considered for eligibility after undergoing a drug washout period. Tapering of the dose of glucocorticoids will be permitted from weeks 4 to 20. An increase in the dose of glucocorticoids is allowed as a rescue therapy once during the trial. No change in non-glucocorticoid immunosuppressants will be permitted. A full list of inclusion and exclusion criteria is provided in Additional file 2.

### Endpoints

Patients will complete the L-PF questionnaire [30] at baseline and then every 4 weeks. This questionnaire, which was developed in patients with pulmonary fibrosis [31], includes a symptoms module (23 questions with recall of the past 24 h) and an impacts module (21 questions with recall of the past week). The symptoms module includes three domains: dyspnoea, cough, fatigue. The primary endpoint in MINT is the change in L-PF questionnaire dyspnoea domain score at week 12.

Key secondary endpoints include the change in L-PF questionnaire dyspnoea domain score at week 24; changes in L-PF questionnaire cough domain, fatigue domain, impacts, and total scores at weeks 12 and 24; the proportion of patients with an increase in dose or change in glucocorticoid/immunosuppressive agent at weeks 12 and 24; and changes in lung function based on clinic PFTs at weeks 12 and 24 (Additional file 2). Safety and tolerability will be evaluated through assessment of adverse events and the proportion of patients who discontinue trial medication.

The rates of patient recruitment, enrolment, screen failure, and drop-out at the local sites and decentralised site will be evaluated. Data collected using remote and clinic enrolment will be compared. The measurement properties of home spirometry versus clinic spirometry will be assessed. Other secondary and exploratory endpoints, including changes in physical activity, steroid use, semi-quantitative and quantitative HRCT scores, pulse oximetry, patient-reported outcomes, and progression-free survival are shown in Additional file 2.

### Sample size

It is planned that 134 patients with progressive MA-ILD will be randomised. In the INBUILD trial in patients with progressive pulmonary fibrosis, the mean (SD) change (increase) from baseline in the L-PF questionnaire dyspnoea domain score at week 12 was 0.59 (13.44) in the nintedanib group and 1.45 (13.04) in the placebo group. Assuming that 134 patients are enrolled in MINT and the drop-out rate is 20%, 108 patients (54 in each treatment group) will have complete follow-up data at week 12. With 108 patients, and assuming a standard deviation of 13.44, the width of a 2-sided 95% confidence interval for the between-group difference in the change in L-PF questionnaire dyspnoea domain score at week 12 in MINT is 2.54, which is considered appropriate for an exploratory study.

### Analyses

Descriptive statistics will be used to summarise the results in each treatment group. No inferential statistics will be performed given the exploratory nature of the study. Efficacy analyses will be performed following an intention-to-treat strategy. Except for safety and tolerability, endpoints at weeks 12 and 24 will be evaluated in patients who have ≥ 1 measurement for the endpoint within the relevant time-frame. Safety and tolerability will be evaluated in patients who receive ≥ 1 dose of trial drug.

## Discussion

MINT is the first large clinical trial to be conducted specifically in patients with MA-ILD. Treatment with nintedanib has been shown to slow decline in lung function in patients with progressive fibrosing ILDs [20, 21, 23, 24]. Acknowledging that the outcomes that are most important to patients with ILDs are those that relate to how they feel and function in their daily lives [32, 33], MINT will investigate the effects of nintedanib on symptoms and quality of life in patients with progressive MA-ILD. Change in the L-PF questionnaire dyspnoea domain score, chosen to be the primary endpoint, has been shown to capture changes in dyspnoea that are meaningful to patients with fibrosing ILDs and to be responsive to changes in disease severity [26, 34].

In conventional clinical trials, the burden associated with clinic visits creates barriers to the recruitment and retention of trial participants. Decentralised trials, in which data are collected remotely, can help reduce these barriers and make trials more patient-centric (Table 1) [35–39]. This is particularly relevant in the case of rare diseases such as MA-ILD, as it eliminates geographical barriers to recruitment. As MINT will utilise both traditional and decentralised recruitment methods, and most study visits will be conducted remotely, the results of this trial will allow an evaluation of the advantages and limitations of decentralised recruitment and remote data collection. They will also enable evaluation of the feasibility and utility of innovative measures such as physical activity monitors and home spirometry. Home spirometry has the potential to increase convenience for patients and provide more frequent measurements than clinic-based spirometry. Moderate to strong cross-sectional correlations between home- and clinic-based measurements have been demonstrated [40–44], but issues with the reliability and variability of home-based measurements have been observed [42, 45, 46]. MINT will be the first trial in patients with ILDs to utilise home spirometry supervised by a technician via telemedicine as well as guided by an avatar, while maintaining adherence to the American Thoracic Society/European Respiratory Society guidelines for clinic-based spirometry [29]. This will provide valuable information that may guide the use of home spirometry in future trials or in clinical practice. To our knowledge, MINT will be the first randomised trial of a treatment for ILD to utilise remote physical activity monitors. Clinical trials in patients with ILDs have usually relied on questionnaires to assess a patient’s physical activity, but physical activity monitors may provide a more accurate measurement of activity levels [47]. The results of MINT will provide information on physical activity levels in patients with MA-ILD and whether these correlate with physiological parameters or patient-reported outcomes.

**Table 1:** Potential advantages and challenges of decentralised trials compared with traditional clinical trials

**Advantages**
Faster recruitment
Expanded geographic reach
Greater diversity and equity of trial participants with respect to race, language, socio-economic status
Lower burden on participants (e.g., travel time and costs)
More patient-centric
Measurements can be more frequent
**Challenges**
A level of digital literacy is required
Participants may feel overburdened with the devices they need to use at home
Additional infrastructure, training and IT support are required
Some patients prefer face-to-face clinical contact

Strengths of MINT include its randomised placebo-controlled design, the enrolment of patients taking standard of care immunosuppressive therapy, the choice of a patient-reported outcome as a primary endpoint, the collection of data on a wide range of endpoints, the use of both local sites and a decentralised site and the mostly remote conduct of the trial. Limitations include a relatively small sample size, short trial duration, and lack of pre-specified statistical testing.

In conclusion, MINT is an innovative hybrid trial that will evaluate the effects of nintedanib on symptoms, quality of life, and ILD progression in patients with progressive MA-ILD and provide valuable information on the utility of decentralised recruitment and remote data collection in clinical trials.

## Supplementary Information

Supplementary Material 1.

Supplementary Material 2.

## Data Availability

The datasets generated and/or analysed during the current study are not publicly available due to the study not being completed but are available from the corresponding author on reasonable request.

## Abbreviations

FVC: Forced vital capacity
HRCT: High-resolution computed tomography
ILD: Interstitial lung disease
IPF: Idiopathic pulmonary fibrosis
JAK: Janus kinase
L-PF: Living with Pulmonary Fibrosis
MA-ILD: Myositis-associated ILD
MINT: Myositis Interstitial Lung Disease Nintedanib Trial
PFTs: Pulmonary function tests
