# Supplementary material for: Design of a randomised controlled hybrid trial of nintedanib in patients with progressive myositis-associated interstitial lung disease
Source: BMC Pulm Med. 2024 Oct 30;24:544. doi: 10.1186/s12890-024-03314-0 (PMC11526615; doi:10.1186/s12890-024-03314-0)

**Supplementary information**

**Additional file 1**

**Supplementary Figure 1.** Recommendations for management of diarrhoea adverse events in the Myositis Interstitial Lung Disease Nintedanib Trial (MINT).


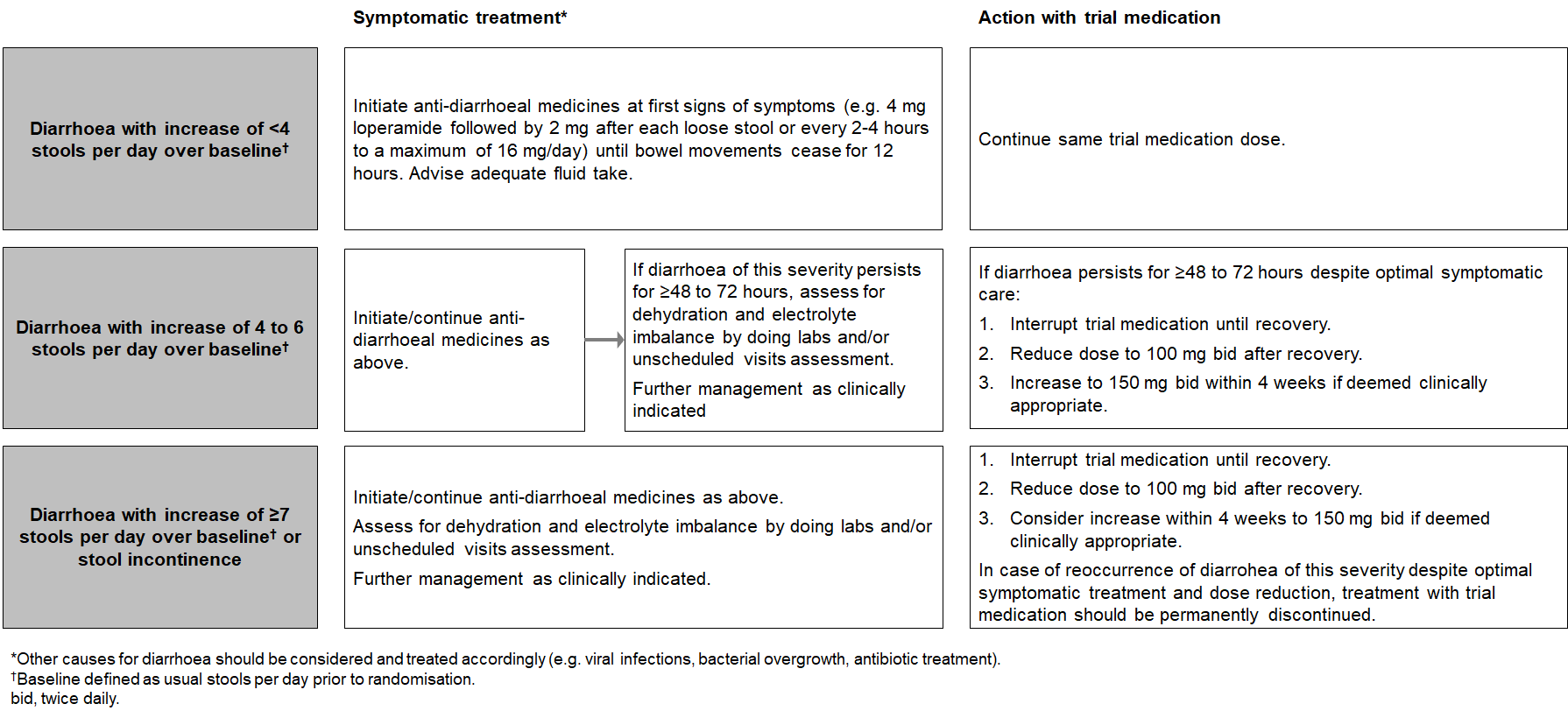


**Supplementary Figure 2.** Recommendations for management of hepatic enzyme elevations in the Myositis Interstitial Lung Disease Nintedanib Trial (MINT).


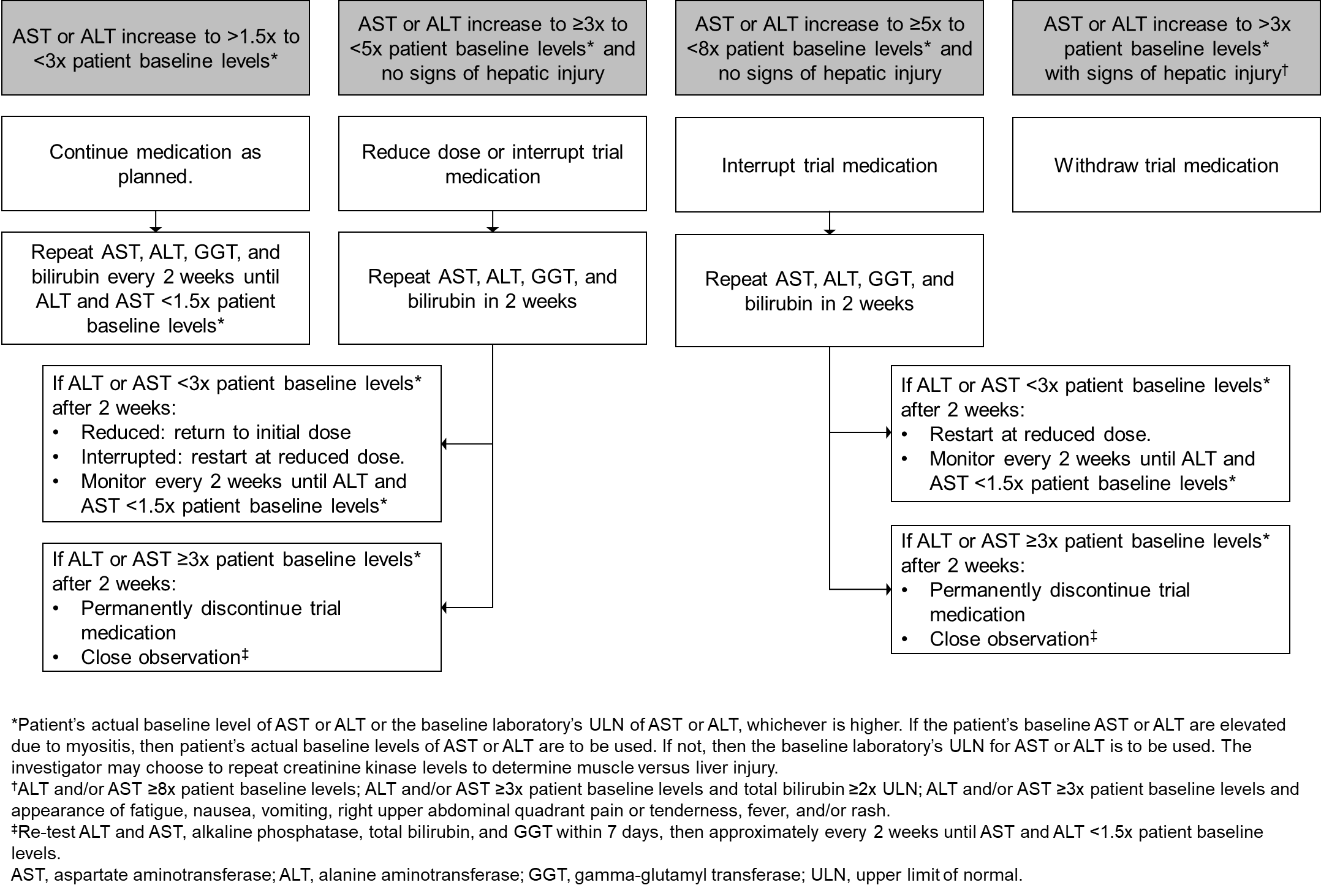

Supplement: Supplementary file 1 — Supplementary Material 1. [file 12890_2024_3314_MOESM1_ESM.docx]
