# Supplementary material for: Design of a randomised controlled hybrid trial of nintedanib in patients with progressive myositis-associated interstitial lung disease
Source: BMC Pulm Med. 2024 Oct 30;24:544. doi: 10.1186/s12890-024-03314-0 (PMC11526615; doi:10.1186/s12890-024-03314-0)
Supplement: Supplementary file 2 — Supplementary Material 2. [file 12890_2024_3314_MOESM2_ESM.docx]

**Supplemental information**

**Additional file 2.**

**Supplementary Table 1.** Inclusion and exclusion criteria of the Myositis Interstitial Lung Disease Nintedanib Trial (MINT)

| **Inclusion criteria** |
| --- |
| Living in USA |
| Age ≥18 years |
| Can speak, read, and understand English or Spanish |
| Willing and capable of performing all study procedures |
| Validity/repeatability of home spirometry confirmed by PFT lab technician/investigator through telemedicine as per ATS/ERS guidelines^1^ |
| Confirmed diagnosis of myositis according to the 2017 ACR/EULAR classification criteria^2^ or by the presence of one of the following autoantibodies:   - Anti-synthetase autoantibody (anti-Jo-1, anti-PL-7, anti-PL-12, anti-EJ, anti-OJ, anti-KS, anti-Tyr, anti-Zo) - Anti-MDA5, TIF1-gamma, Mi-2, NXP2/MJ, SAE, HMGCR, SRP - Anti-PM/Scl, Ku, U1RNP, Ro5,2/60 or SSA (in absence of clinical diagnosis of systemic sclerosis or primary Sjogren syndrome) |
| Fibrosing interstitial lung disease:   - Chest HRCT ≤12 months before screening with fibrosing ILD (reticular changes, traction bronchiectasis and/or honeycombing) - No other identifiable cause of fibrosis - The following co-existing features are accepted: ground glass opacity, upper lung or peri-bronchovascular predominance, mosaic attenuation, air trapping, consolidation, centrilobular nodules |
| Progressive ILD, defined as meeting ≥1 of the following criteria at any time within the 24 months before screening   - ≥10% relative decline in FVC% predicted - ≥5 to <10% relative decline in FVC % predicted with worsened dyspnoea - ≥5 to <10% relative decline in FVC % predicted with worsened fibrosis on chest HRCT - Worsened dyspnoea with worsened fibrosis on chest HRCT |
| FVC >40% predicted and ≤80% predicted |
| Standard of care therapy:   - 1 glucocorticoid and 1 non-glucocorticoid immunosuppressive medication *or* 2 non-glucocorticoid immunosuppressive medications - The immunosuppressive component must have been started ≥12 weeks prior to, and be stable for ≥4 weeks prior to, the screening visit - The glucocorticoid component must have been started ≥4 weeks prior to, and be stable for ≥2 weeks prior to, the screening visit - Allowable immunosuppressive medications and glucocorticoids:   - Glucocorticoid (maximum dose ≤20 mg/day prednisone equivalent)   - Mycophenolate mofetil (maximum dose 3 gm/day)   - Mycophenolic acid (maximum dose 1440 mg/day)   - Azathioprine (maximum dose 2.5 mg/kg/day)   - Methotrexate (maximum dose 25 mg/week)   - Tacrolimus (maximum dose 10 mg/day)   - Cyclosporine (maximum dose 200 mg/day)   - Leflunomide (maximum dose 20 mg/day)   - Sulfasalazine (maximum dose 3 gm/day)   - Intravenous or subcutaneous immunoglobulin (maximum dose 2 gm/kg/month) (not considered immunosuppressive therapy)   - Rituximab if given ≥4 weeks before screening   - Hydroxychloroquine is allowed and not considered immunosuppressive therapy   - JAK inhibitors such as tofacitinib, baricitinib, upadacitinib - Inhaled medication(s) for lung disease is allowed if started >4 weeks before screening (These should remain stable throughout the trial) |
| Negative pregnancy test for women, and men and women of reproductive potential must agree to use 2 reliable methods of birth control |
| **Exclusion criteria** |
| Planned major surgical procedures within trial period |
| Women who are pregnant, nursing, or who plan to become pregnant while in the trial |
| Severe lung disease defined by the following ≤6 months before screening:   - FVC ≤40% predicted - DLco <30% predicted (corrected for haemoglobin) - Oxygen ≥10L at rest based on home oxygen prescription - Listed or under evaluation for lung transplant |
| Moderate to severe active muscle disease from myositis as per any one of these criteria:   - Creatine kinase >1000 U/mL - Moderate to severe dermatomyositis rashes as per investigator evaluation (if rash present) - Moderate to severe arthritis as per investigator evaluation - Moderate to severe muscle weakness as per 30-second Sit to Stand test <7 |
| History of or ongoing serious active, chronic, or recurrent infection ≤4 weeks before screening |
| Significant pulmonary hypertension defined by any of the following:   - Previous diagnosis of moderate to severe pulmonary hypertension or significant right heart failure - History of echocardiographic evidence of significant right heart failure or moderate to severe pulmonary hypertension (TR jet ≥2.9 m/s and signs of RV dysfunction; TR jet >3.4; RVSP >40 to 55 with evidence of RV strain or dysfunction; RVSP >55) - History of right heart catheterization showing a cardiac index ≤2.2 l/min/m² or mPAP >40 mmHg with a PCWP <15 mmHg - Pulmonary hypertension requiring oral, IV, or inhaled therapy (such as epoprostenol, treprostinil, iloprost, bosentan, ambrisentan, sildenafil, tadalafil) |
| Increased bleeding risk defined by any of the following:   - Patients who require:   - Fibrinolysis, full-dose therapeutic anticoagulation (e.g. vitamin K antagonists, direct thrombin inhibitors, heparin, factor Xa inhibitors, low molecular weight heparin)   - High-dose antiplatelet therapy (>325 mg acetylsalicylic acid or >75 mg clopidogrel) - Haemorrhagic central nervous system event ≤12 months before screening - Either of the following ≤3 months before screening:   - Haemoptysis or haematuria   - Active gastrointestinal bleeding or active gastrointestinal ulcers - Coagulation parameters: INR >2, prolongation of PT and aPTT by >1.5 x ULN at screening |
| Thrombotic event (including stroke and transient ischemic attack) ≤12 months before screening |
| Severe cardiovascular disease defined by any of the following:   - Severe hypertension, uncontrolled under treatment (≥160/100 mmHg) ≤6 months before screening - Myocardial infarction or unstable cardiac angina ≤6 months before screening |
| Chronic liver disease (Child-Pugh A, B, or C) |
| Known hypersensitivity to trial medication or its components (i.e. soya lecithin) |
| Other diseases that may interfere with testing procedures or, in the judgment of the investigator, may interfere with trial participation, or may put the patient at risk when participating in this trial, or significant other lung diseases |
| Life expectancy for a disease other than ILD <2.5 years |
| Other investigational therapy ≤1 month or 6 half-lives (whichever was greater) before screening |
| Previous treatment with nintedanib or pirfenidone (taken for ≥1 month or history of intolerance/side effects) |
| Current or recent use of one or more of the following medications:   - Cyclophosphamide ≤3 months before randomisation - Rituximab ≤4 weeks before randomisation - Anti-TNF (infliximab, golimumab, or certolizumab) ≤8 weeks or adalimumab within ≤4 weeks, and etanercept ≤2 weeks before randomisation - Anakinra ≤1 week before randomisation - Other biological agents such as tocilizumab, abatacept, ≤4 weeks before randomisation |
| Safety laboratory abnormality:   - AST and/or ALT >1.5 x ULN, unless deemed due to active myositis, in which case creatinine kinase is also abnormally elevated and the ratio of AST or ALT by creatinine kinase levels (adjusted as x ULN) should be <2.0 and GGT <2.0 x ULN - Bilirubin >1.5 x ULN - Creatinine clearance <30 mL/min (Cockcroft–Gault formula) - Haemoglobin <9.0 - Platelet count <100,000/mm^3^ - White blood cell count <3000/mm^3^ |

1. Graham BL, et al. Standardization of spirometry 2019 update. An official American Thoracic Society and European Respiratory Society technical statement. Am J Respir Crit Care Med 2019;200:e70-e88. 2. Lundberg IE, et al. 2017 European League Against Rheumatism/American College of Rheumatology classification criteria for adult and juvenile idiopathic inflammatory myopathies and their major subgroups. Arthritis Rheumatol 2017;69:2271-2282.

ACR, American College of Rheumatology; ALT, alanine transaminase; aPTT, activated partial thromboplastin time; AST, aspartate transaminase; ATS, American Thoracic Society; DLco, diffusing capacity of the lungs for carbon monoxide; ERS, European Respiratory Society; EULAR, European Alliance of Associations for Rheumatology; FEV_1_, forced expiratory volume in 1 second; FVC, forced vital capacity; GGT, gamma-glutamyl transferase; HRCT, high-resolution computed tomography; ILD, interstitial lung disease; INR, international normalised ratio; JAK, janus kinase inhibitor; mPAP, mean pulmonary arterial pressure; PCWP, pulmonary capillary wedge pressure; PFT, pulmonary function test; RV, right ventricular; RVSP, right ventricular systolic pressure; TNF, tumour necrosis factor; TR, tricuspid regurgitant; ULN, upper limit of normal.

**Supplementary Table 2.** Standard of care therapies permitted in the Myositis Interstitial Lung Disease Nintedanib Trial (MINT).

| **Therapy** | **Maximum dose** |
| --- | --- |
| Glucocorticoid | 20 mg/day prednisone or equivalent |
| Mycophenolate mofetil | 3 g/day |
| Mycophenolic acid | 1440 mg/day |
| Azathioprine | 2.5 mg/kg/day |
| Methotrexate | 25 mg/week |
| Tacrolimus | 10 mg/day |
| Cyclosporine | 200 mg/day |
| Leflunomide | 20 mg/day |
| Sulfasalazine | 3 g/day |
| Intravenous or subcutaneous immunoglobulin | 2 g/kg/month |
| Rituximab | Not specified |
| JAK inhibitors | Not specified |

**Supplementary Table 3.** Key and other secondary endpoints of the Myositis Interstitial Lung Disease Nintedanib Trial (MINT).

| **Key secondary endpoints** |
| --- |
| Change in L-PF questionnaire dyspnoea domain score at week 24 |
| Changes in L-PF questionnaire cough and fatigue domain scores at weeks 12 and 24 |
| Changes in L-PF questionnaire symptoms, impacts and total score at weeks 12 and 24 |
| Proportion of patients with increase in dose or change in glucocorticoid / immunosuppression agent for clinical worsening/flare of MA-ILD at weeks 12 and 24 |
| Lung function based on clinic PFTs:   - Absolute and relative changes in FVC (mL and % predicted) at weeks 12 and 24 - Proportions of patients with a relative decline in FVC (mL and % predicted) of ≥10%, ≥7.5% and ≥5% at weeks 12 and 24 - Proportions of patients with stable (± <5%) or improved (≥5, ≥7.5, ≥10%) FVC (mL and % predicted) at weeks 12 and 24 - Times to improvement and decline in FVC (mL and % predicted) of ≥5%, 7.5%, 10% over 24 weeks - Time to progression defined as:  1. Decline in FVC (mL) ≥10%, death or lung transplant over 24 weeks 2. ILD worsening (any of the following after 4 weeks: decline in FVC % predicted ≥30%; HRCT suggesting severe worsening; hospitalisation with severe ILD with new oxygen requirement related to worsening ILD; severe worsening in dyspnoea due to ILD), death or lung transplant over 24 weeks 3. Non-elective hospitalisation for ILD worsening/flare, death or lung transplant over 24 weeks |
| **Other secondary endpoints** |
| Rate of patient recruitment, enrolment, screen failure, and drop-out rates between the local sites (average) and the remote site |
| Psychometric properties of home spirometry compared to clinic/lab-based spirometry |
| Absolute and relative changes in DLco and FEV_1_, and thresholds of change similar to FVC (see above) |
| Progression-free survival   - Death, or lung transplant, or relative decline in FVC % predicted ≥10%, or relative decline in FVC % predicted ≥5% with relative decline in DLco % predicted ≥15% evaluated at weeks 12 and 24 - Clinical worsening is defined as death, lung transplant, new/worsening oxygen use at rest (>2 L), non-elective hospitalisation for ILD clinical worsening/flare or ILD worsening (any one of the following after 4 weeks of treatment: decline in FVC % predicted ≥30%; HRCT suggesting severe worsening; hospitalisation with severe ILD with new oxygen requirement related to worsening ILD; severe worsening in dyspnoea due to ILD), or out-of-protocol rescue medication for ILD clinical worsening/flare |
| Steroid use (calculated using prednisone dose equivalents) at weeks 12 and 24:   - Mean and median decrease in glucocorticoid dose - Absolute and relative changes in glucocorticoid dose - Area under the curve for cumulative glucocorticoid exposure - Proportion of patients who decreased glucocorticoid to 5, 7.5, and 10 mg or less of prednisone equivalent - Proportion of patients receiving glucocorticoid rescue therapy (within protocol) for ILD clinical worsening/flare - Proportion of patients requiring intravenous glucocorticoid rescue therapy for ILD clinical worsening/flare (resulting in withdrawal of trial drug) |
| Change in supplemental oxygen needs from baseline to weeks 12 and 24 (captured via items in L-PF questionnaire symptoms score) |
| Overall assessments of change from baseline to weeks 12 and 24:   - Patient overall assessment of change - Physician assessment of change |
| Changes in physical activity monitor (step-based and cadence measures) |
| Changes in sit-to-stand test |
| Changes in HRCT from baseline to week 24 using semi-quantitative and quantitative image-based scoring |
| Adverse events and tolerance:   - Proportions of patients with any adverse events, serious adverse events, and adverse events of special interest (gastrointestinal perforation, hepatic injury) - Proportion of patients who discontinue study drug |

DLco, diffusing capacity of the lungs for carbon monoxide; FEV_1_, forced expiratory volume in one second; FVC, forced vital capacity; HRCT, high-resolution computed tomography; L-PF, Living with Pulmonary Fibrosis; MA-ILD, myositis-associated interstitial lung disease.

**Supplementary Table 4.** Exploratory endpoints of the Myositis Interstitial Lung Disease Nintedanib Trial (MINT).

| Lung function based on home spirometry:   - Absolute and relative changes in FVC (mL and % predicted) at weeks 12 and 24 - Proportions of patients with a relative decline in FVC (mL and % predicted) of ≥10%, ≥7.5% and ≥5% at weeks 12 and 24 - Proportions of patients with stable (± <5%) or improved (≥5, ≥7.5, ≥10%) FVC (mL and % predicted) at weeks 12 and 24 - Times to improvement and decline in FVC (mL and % predicted) of ≥5%, 7.5%, 10% over 24 weeks - Time to progression defined as:  1. Decline in FVC (mL) ≥10%, death or lung transplant over 24 weeks 2. ILD worsening (any of the following after 4 weeks: decline in FVC % predicted ≥30%; HRCT suggesting severe worsening; hospitalisation with severe ILD with new oxygen requirement related to worsening ILD; severe worsening in dyspnoea due to ILD), death or lung transplant over 24 weeks 3. Non-elective hospitalisation for ILD worsening/flare, death or lung transplant over 24 weeks |
| --- |
| Changes in other patient-reported outcomes:   - St. George’s Respiratory Questionnaire (SGRQ) - Patient-Reported Outcome Measurement Information System (PROMIS) physical function-20 - Grading of myositis symptoms |
| Changes in muscle enzymes (creatinine kinase, aldolase, AST, ALT) |
| Changes in pulse oximetry |
| Patient’s perspective of the trial |

ALT, alanine transaminase; AST, aspartate transaminase; FVC, forced vital capacity; HRCT, high-resolution computed tomography; ILD; interstitial lung disease.
